# From Barriers to Solutions: A Community Network Approach to Equitable Oral Healthcare Access

**DOI:** 10.1016/j.identj.2026.109751

**Published:** 2026-07-14

**Authors:** Sehida Begovic, Michiel W. van der Linden, Sterre J. Gitz, Stefan Listl, Linnea Eisemann de Almeida, Esben Boeskov Øzhayat, Kasper Rosing, Catherine Volgenant, Monique H. van der Veen

**Affiliations:** a Department of Oral Public Health, Academic Centre for Dentistry Amsterdam, University of Amsterdam and VU University Amsterdam, The Netherlands; b Department of Cariology, Academic Centre for Dentistry Amsterdam, University of Amsterdam and VU University Amsterdam, The Netherlands; c Section for Oral Health, Heidelberg Institute of Global Health, Heidelberg University Hospital, Heidelberg, Germany; d Department of Dentistry, Quality and Safety of Oral Healthcare, Radboud University Medical Center–Radboud Institute for Health Sciences (RIHS), Nijmegen, the Netherlands; e Section for Oral Health, Society and Technology, Department of Odontology, University of Copenhagen, Denmark; f Department of Preventive Dentistry, Academic Centre for Dentistry Amsterdam, University of Amsterdam and VU University Amsterdam, The Netherlands; g Department of Paediatric Dentistry, Academic Centre for Dentistry Amsterdam, University of Amsterdam and VU University Amsterdam, The Netherlands

**Keywords:** Community, Oral health, Social work, Interdisciplinary health, Quality of care, Access to oral care

## Abstract

**Introduction:**

Across Europe, oral health is increasingly recognized as a public health priority. Still, structural, financial, and social barriers disproportionately affect people in vulnerable circumstances.

**Objective:**

Within the EU-funded DELIVER project, this study aimed to (1) identify which barriers to oral healthcare manifest across urban contexts, and (2) explore how community networks including citizens, healthcare professionals, social workers, policy makers, and other local stakeholders can contribute to developing local solutions related to improving quality of oral healthcare for citizens in vulnerable circumstances.

**Methods:**

A participatory action research design, guided by the Community Health Improvement Process, was used. Participants included citizens with lived experience of poverty or social exclusion, oral healthcare professionals, social workers, and policymakers. Data collection methods included semi-structured interviews, focus groups, co-creation meetings, and creative workshops. Data were analyzed using inductive thematic analysis.

**Results:**

A total of 64 participants contributed to 21 interviews, 7 focus groups, and 4 co-creation meetings. Seven key barriers were identified: high treatment costs, limited insurance coverage, low oral health literacy, emotional and psychological factors (e.g., shame and fear), competing life priorities, limited support from professional organizations, and poor communication between social and oral healthcare services. Proposed solutions included walk-in consultations in community settings, buddy systems, improved support for navigating insurance, stronger integration of dental and social care, and oral health promotion through trusted local networks.

**Conclusion:**

Community-network approaches can substantially reduce inequalities in access to oral healthcare. Engaging citizens as co-creators enables inclusive and needs-based solutions and improvements. This study offers a promising model for improving oral healthcare accessibility by systematically identifying barriers and addressing them through community-based collaboration.

## Introduction

Oral health problems continue to contribute to the global burden of disease, despite the preventable nature of most oral diseases through adequate oral hygiene, fluoride use, and timely preventive care.^1^^,^^2^ Even in high-income regions such as the European Union, dental caries, periodontal diseases, and tooth loss remain prevalent, particularly among individuals from lower socio-economic backgrounds.^3^ According to 2024 data from the Organization for Economic Co-operation and Development (OECD), dental care is among the most frequently unmet healthcare needs in the European Union due to financial barriers, with avoidance of oral healthcare being particularly pronounced among individuals in the lowest income quintile: for example, for the Netherlands, 1.1% in the lowest income quantile avoided oral healthcare for financial reasons vs. 0,6% on average.^4^ There is a lack of research exploring collaborative approaches that actively involve citizens, healthcare professionals, social workers, policy makers, and other local stakeholders in improving access to oral healthcare for people in vulnerable circumstances. Addressing this gap is important to better understand how locally embedded networks may support the development of more inclusive and accessible oral healthcare systems.

The DELIVER project (DELiberative ImproVEment of oRal care quality) aims to develop a blueprint model for improving the quality of oral healthcare for everyone.^5^ The project was initiated to support the translation of deliberative dialogues into actionable solutions for improving oral healthcare at community, practice, and national/international levels. Early project phases involved identifying and prioritizing pressing issues in oral healthcare quality through deliberative sessions with researchers, policymakers, healthcare providers, and patients/citizens. These stakeholders consistently ranked accessibility and affordability among the most urgent issues to address.^6^

At the community level, the DELIVER project has mapped out the wide range of stakeholders that citizens in vulnerable circumstances interact with when they seek access to oral healthcare.^7^ Generally, those with the highest need for healthcare are least likely to have access to it. This so-called ‘inverse care law’^8^ remains highly relevant in oral healthcare: those with the highest need are least able to obtain care, particularly individuals experiencing poverty, limited insurance coverage or social exclusion, especially in systems where adults have to pay for care out-of-pocket (Begovic & Chau et al., 2025).^9^ Chronic stress and social challenges, including poor housing, food insecurity and unstable living conditions, also affect oral self-care and health behaviors.^10^

Co-creation has been proposed as a promising approach to address healthcare barriers within communities. Co-creation involves collaborative development of solutions by patients, communities and professionals, emphasizing shared problem-solving, mutual learning and alignment with local needs.^11^ By involving end-users in design and implementation, co-creation uncovers nuanced barriers (e.g., cultural, economic) that quantitative data might miss. Co-creation creates trust and ownership, increasing the likelihood of sustained adoption. Continuous feedback loops allow iterative adjustments to reflect evolving needs.^11^^,^^12^

Various community health initiatives illustrate the potential of community-based collaboration. Community Health Worker programs, for instance, have demonstrated benefits for quality of care through direct support, health education, and improved navigation of health systems.^13^ An example of a community health initiative in the Netherlands is a collaboration between dental practitioners and social organizations (the Social Dentist initiative featuring a National Dentists’ Day), where dental care is offered to people living in poverty.^14^ Such initiatives, in which care is provided to people with limited access to services, are often focused on delivering emergency treatment, for example, relieving pain, and are less oriented towards sustainable, long-term solutions to improve the quality of oral healthcare for people in vulnerable circumstances.

To achieve sustainable quality improvements, broader community collaboration is required. Establishing community networks that bring together oral healthcare providers, policymakers, social workers, NGOs, and community health professionals may help integrate oral health into wider social and public health structures.^7^ Participatory action approaches have shown promise for engaging underserved communities, identifying context-specific access barriers, and developing solutions grounded in local realities (Agnello et al., 2025; Greenhalgh et al., 2016).

This study, part of the DELIVER project, aimed to (1) identify which barriers to oral healthcare manifest across urban contexts, and (2) explore how community networks including citizens, healthcare professionals, social workers, policy makers and other local stakeholders can contribute to developing to local solutions related to improving quality of oral healthcare for citizens in vulnerable circumstances.

## Methods

### Study design and CHIP-model

This study employed a community-based participatory action research (PAR) design in the Netherlands, using a co-creation approach to collaboratively develop community-level strategies for improving oral healthcare accessibility and delivery.^11^^,^^15^ Citizens with lived experience, oral healthcare professionals, social workers and policy makers were actively involved throughout the research process as collaborative stakeholders. The study consisted of multiple iterative phases, including stakeholder engagement, problem identification, priority setting, and collaborative solution development. Data collection included semi-structured interviews and focus group discussions. Findings from the semi-structured interviews and first focus group discussions informed subsequent co-creation activities and action planning. The overall process was guided by the principles of the Community Health Improvement Process (CHIP) framework,^16^ which structures collaborative efforts around community health assessment, stakeholder engagement, priority setting, and actionable planning, as described in detail elsewhere.^17^ In line with this framework, the study followed 2 interconnected cycles: (1) a Problem Identification and Prioritization Cycle, during which a community coalition was established and key oral healthcare challenges were identified, and (2) an Analysis and Implementation Cycle, during which stakeholders collaboratively developed and implemented potential strategies for improvement.^16^

### Ethical considerations

The research protocol for this study was deemed not subject to the Dutch Medical Research Involving Human Subjects Act by the Academic Centre for Dentistry Amsterdam Institutional Review Board (ACTA-ETC protocol No. 2023-26070). The study was conducted in accordance with the ethical principles of the Declaration of Helsinki, including voluntary participation, written informed consent, confidentiality, and the right to withdraw at any time without consequences. Anonymity and confidentiality were safeguarded throughout data analysis. Participants were offered lunch or snacks during sessions and were informed that they could withdraw at any point without any consequences.

### Invitations of participants

Citizens, (oral) healthcare professionals, social workers, and policy makers were considered eligible, based on prior research wherein we explored which key actors were involved in community-level quality improvement of oral healthcare.^7^ Dutch cities show higher poverty rates than more rural areas. In 2023, these rates were 6.6% and 6.2% for Amsterdam and Rotterdam, respectively, as compared to an average of 3.1% in the Netherlands.^18^ Therefore, further eligibility criteria for citizens included current residence in Amsterdam or Rotterdam and self-reported experiences of vulnerability, such as homelessness, poverty, or low income. Recruitment took place primarily in community centers using an open and inclusive strategy designed to invite diverse group of participants. Citizens were recruited using a combined convenience- and snowballing sampling strategy^19^^,^^20^ through local community centers, social services, and neighborhood networks. Healthcare professionals, social workers, and policy makers were primarily recruited through convenience sampling using existing professional networks that the researchers had developed close associations with in a previously performed Situational Analysis in Amsterdam,^7^ while in Rotterdam established professional networks were approached to recruit additional participants through snowball sampling. To reduce potential selection bias associated with convenience and snowball sampling, recruitment was conducted through diverse community organizations, social services, and professional networks in both cities, aiming to include participants with diverse professional backgrounds and lived experiences. In addition, citizens were not grouped together with their own healthcare or social care professionals during data collection, to facilitate open discussion.

### Data collection

Data were collected through semi-structured interviews, focus group discussions (FGDs), and co-creation sessions. Individual interviews and FGDs were audio-recorded. The semi-structured interviews explored 6 sections: participants’ experiences with oral health and oral healthcare, perceptions of care quality and accessibility, healthcare-seeking behaviors, experiences with oral health professionals, barriers and facilitators to care, and preferences for participation in co-creative oral healthcare improvement initiatives. Preliminary topic guides for the FGDs were jointly developed by researchers from ACTA and the University of Copenhagen. The focus group discussions were guided by the central question: *“How can citizens and professionals collaborate at the community level to improve oral healthcare delivery and accessibility?”* The discussions explored participants’ experiences with dental care, including perceived barriers, challenges, and positive aspects of oral healthcare access and delivery within their community. In addition, participants discussed priorities for improvement, opportunities for collaboration between citizens and professionals, expectations regarding involvement in co-creative activities, and conditions that could support community engagement in improving oral healthcare accessibility and quality. Each FGD informed the design and focus of the subsequent sessions. Insights and priorities identified by participants were discussed collectively and used to shape the topics, questions, and format of the next FGD. This iterative process reflected the adaptive and co-creative nature of the research.

### Participatory methods

In addition to verbal discussions, we incorporated multiple creative and participatory methods to facilitate inclusive engagement, shared decision-making, and active participation of citizens with diverse communication and literacy levels. Poster exercises, involving the use of sticky notes for group brainstorming, were used to identify needs and barriers and to collaboratively develop themes. Participants first wrote individual ideas on sticky notes, which were then grouped and discussed collectively, supporting co-creation, prioritization, and shared reflection. Story-telling conversations were used to stimulate reflection on lived experiences and to collaboratively define aspirational future scenarios ("dream situations") regarding oral healthcare. Meetings were intentionally organized in community locations chosen together with participants to create accessible and low-threshold environments for participation. To support relationship-building and engagement, sessions also incorporated energizers (short playful exercises and games), informal shared meals, and dedicated moments for oral health-related questions from citizens concerning fluoride use, self-care, and preventive dental practices. These activities aimed to foster trust, social cohesion, empowerment, and reciprocal knowledge exchange between citizens and professionals.

### Data analysis

Audio-recordings were transcribed verbatim in Microsoft Word (Microsoft Corporation, Redmond, WA, USA). All transcripts and analyses were conducted in the original language (Dutch) to preserve contextual meaning and nuance, quotations were translated into English only during manuscript preparation. Qualitative data were analyzed using ATLAS.ti (version 25) (ATLAS.ti Scientific Software Development GmbH, Berlin, Germany) and thematic analysis was used to identify barriers to oral healthcare, shared experiences, and community-level patterns.^21^ One researcher (SB) coded transcripts, after which coding frameworks were compared and refined through team discussions and member checked by participants from the focus group discussions.

### Researcher reflexivity

Decisions throughout the co-creation process were made collectively, with final decisions grounded in citizen input and needs. Efforts were made to ensure participants were not merely sources of data but co-creators of knowledge and drivers of change. The research team held regular sessions to reflect on this. During reflection sessions, positionality, power dynamics, and potential biases in analysis and facilitation were examined as described elsewhere.^15^^,^^22^ These reflections focused on how researchers’ social positions, professional backgrounds, and lived experiences might have influenced interactions with participants and the interpretation of findings. Attention was also given to power dynamics within the research process as we had different professionals and citizens participated together, aiming to create a more equitable space for participants’ voices and perspectives to shape the outcomes of the study.

### Validation and community accountability

The co-creation process was iterative. A continuous process of member checking was used, in which preliminary findings were returned to citizen participants in feedback sessions to refine and enhance them.^23^ These sessions enabled participants to validate interpretations, correct misrepresentations, and influence subsequent steps. This ongoing feedback loop reinforced the collaborative ethos of the research and ensured the findings were deemed relevant by those involved.

## Results

As part of the DELIVER-project, 3 urban community networks were established in Amsterdam-Noord, Amsterdam-Zuidoost, and Rotterdam. In total, 64 unique individuals contributed to the study, with some participating in both interviews and FGDs.

*Individual interviews:* A total of 21 semi-structured interviews were conducted with participants from Rotterdam (N = 11) and Amsterdam (N = 10). These included 14 citizens and 7 professionals, comprising 2 oral healthcare (OHC) providers, 3 social workers, 1 financial debt administrator, and 1 program manager from a local social foundation.

*Co-creation sessions:* In addition, 7 focus group discussions (FGDs) and 4 co-creation sessions were conducted with 47 participants (some attended more than one session), including 25 citizens and 22 professional stakeholders. Among the professionals were 3 national policymakers, 4 local policymakers, 2 OHC professionals, and 13 social workers.

### Identified needs and barriers

Following the first stage of the CHIP-model,^16^ problems were identified and prioritized. Results from the interviews and focus group discussions revealed a wide range of interconnected barriers to oral healthcare access among citizens in vulnerable circumstances. These barriers were grouped into seven overarching themes, each comprising multiple needs relevant to the theme/barrier: (1) financial barriers, (2) health insurance barriers, (3) knowledge and health attitudes, (4) emotional and psychological barriers, (5) competing priorities, (6) social support and (7) communication and behaviour from OHC providers.

**Fig. 1:**
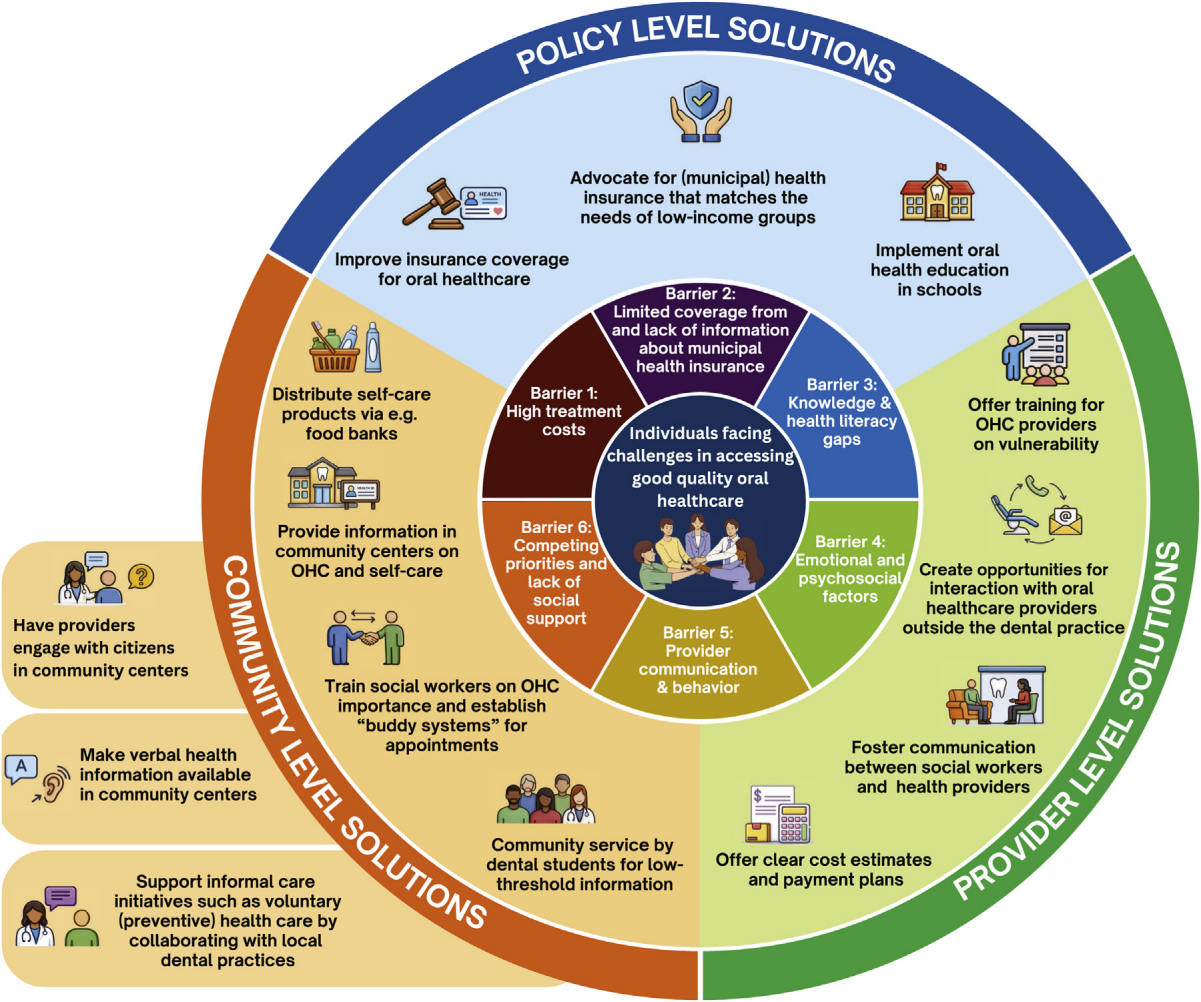
Key barriers and corresponding solutions for quality improvement of oral healthcare across the policy, practice, and community levels.

In addition to identifying challenges, participants actively proposed contextualized and feasible solutions for each barrier. Feedback and reflection were continuously incorporated in the process. Table 1 provides an overview of the identified themes, subthemes, and proposed solutions. Figure 1 presents a summary of the key barriers and corresponding solutions across the policy, practice, and community levels.

**Table 1:** Key challenges and proposed solutions for better oral healthcare for people in vulnerable circumstances.

Barriers/needs (Themes)	Key challenges (Sub-themes)	Proposed solutions
**Financial barriers**	• High treatment costs for both employed and unemployed citizens • Expensive self-care products • Dental care perceived as luxury • Difficult to estimate treatment costs	• National policy changes: coverage for OHC from basic health insurance for people with low-income • Provision of self-care products through municipalities, food banks or health insurers. • Better information about dental healthcare costs available in communities (dentist-walk-in-moments) • Promoting informal care initiatives such as the Social Dentist • Dentists offering payment plans in installments
**Health insurance barriers**	• Very limited coverage from basic health insurance • Health insurance system difficult to navigate • Unclarity about coverages • Not all necessary treatments are covered • Limited coverage from collective health insurance from municipalities • Lack of information about collective health insurance packages from municipalities	• National policy changes: coverage for OHC from basic health insurance for people with low-income • Information about navigating health insurances available in community center, specifically targeted at oral healthcare coverage. • Advocating for better collective health insurance from municipalities • Advocating for political awareness regarding OHC accessibility limitations.
**Knowledge and health attitudes**	• Low health literacy among people in vulnerable circumstances • Lack of preventive routines from childhood • Complex healthcare systems • Unfamiliarity with availability of local support resources • OHC information not available outside of oral healthcare practices • Unfamiliarity with criteria for choosing a new dentist (e.g. willingness to treat people with autism or anxiety)	• OHC education in schools • Make verbal information instead of written information available in community centers • Ask dental associations to provide a map of dentists who are willing to provide care for vulnerable people • Include dental students to provide community service in neighborhood locations
**Emotional and psychological barriers**	• Shame about oral health condition and social situation (vulnerability) • Stigma of fear and judgement • Dental anxiety • Traumatic past experiences • General distrust in dentists (suspicion of commercial motives)	• Opportunities to discuss OHC with dental practitioners outside of dental practices for better mutual understanding (community service)
**Competing priorities**	• Survival stress (poverty, housing issues, mental health problems) • OHC not structurally integrated in general or social care • Social workers face more urgent issues leaving little time for OHC	• Training program for social workers to create more awareness about OHC
**Social support**	• Lack of professional social support regarding OHC • Lack of social support among friends (e.g. friends in similar situations, not possible to borrow money) • Lack of financial resources to maintain a social network	• Buddy-system in community centers to help with appointments, transportation and understanding OHC information provided by dentists.
**Communication and behaviour from OHC providers**	• Need for good arrangements with dentists about costs or anesthesia • OHC professionals provide conflicting information about oral health or products • No collaboration between OHC providers and general practitioners • Limited space within consultations to ask questions or discuss financial issues • Ice-breaker needed before treatment	• OHC professionals should create a low threshold, e.g. by going into communities, engaging with citizens in neighborhood centers • Providing OHC information in GP offices • Training program for OHC professionals about vulnerability

*1. Financial barriers*

Financial barriers were discussed by nearly all participants across interviews and focus group discussions. *“And I know I’m not the only one [who cannot afford the dentist]. I have hardworking friends with full-time jobs who still haven’t been able to visit the dentist. One of my friends and her boyfriend both work 40 hours a week, yet they still can’t afford the costs. Her boyfriend is missing a front tooth, he’s a car mechanic, and she works for a large shipping company. How bad is that?”* – *Rotterdam, individual interview 10, citizen who received informal no-cost dental care.*

Self-care products such as toothbrushes, toothpaste, and fluoride rinses were also described as unaffordable for some. Many participants perceived dental care as a luxury service, not an essential part of general health: *“Sometimes it feels as if dental care is a luxury product. If you have a low income or are in a difficult situation, it’s as if you don’t have the right to a beautiful smile. Yet teeth are so important for your overall health. Dental care really needs to become much more accessible.”* – *Rotterdam, individual interview 9, citizen who received informal no-cost dental care.*

The lack of transparency around treatment costs made it difficult for participants to anticipate expenses in advance, further discouraging care-seeking behaviour. In addition, citizens expressed a need for oral healthcare providers to offer guidance in navigating available resources when individuals are unable to afford the costs of dental care: *“No one ever told me that I could go to a social worker if I couldn’t afford the dentist. Usually, when you have a toothache, you just go to the dentist. But if you tell the dentist you don’t have any money, they just send you home again. I think the dentist should refer you to a social worker.”- Amsterdam, individual interview 1, recipient emergency fund.*

*2. Health Insurance Barriers*

Citizens expressed confusion and dissatisfaction regarding the limited coverage of oral healthcare in both basic health insurance packages available and mandatory for everyone and collective municipal insurance packages available through municipalities for people with low-income: *“I still don’t understand. They [social worker] told me I could switch insurance and that 80% of dental costs would be covered, up to 500 euros. But that’s confusing. Just say you’ll pay 500 euros. I’m starting to understand it a bit more, but it’s still vague to me: why do you have a premium, then extra, and extra again, and why does one thing cover this but not that?” – Amsterdam, individual interview 3, recipient emergency fund.* Many found the insurance system difficult to navigate, and there was widespread uncertainty about what is and isn’t covered, including common procedures such as fillings or extractions. Even collective plans offered by municipalities were described as insufficient or poorly communicated: *“We would like information about the collective Amsterdam insurance, but also about general health insurance, because many Amsterdam residents are not part of the collective scheme. That’s a major problem, since every insurer has its own rules. You should be able to know whether there are national regulations requiring certain reimbursements without extra costs. But you can’t find this information anywhere. Maybe each insurer should have a dedicated department to provide such information, also for dental care.”* – *Amsterdam, FGD2, citizen-participant 3*

*3. Knowledge and health attitudes*

Lack of knowledge about oral health and the absence of early-life preventive routines were described as structural problems among many citizens: *“Because of the poor care I received from my parents, my teeth are in bad condition. I often had to have teeth pulled, and those negative experiences made me lose motivation to seek dental care.”- Rotterdam, individual interview 13, citizen who received informal no-cost dental care. ‘’What makes a toothpaste good to use and how can you see whether it contains fluoride?’’ – Rotterdam, FGD1, citizen-participant 1.* Participants noted that oral healthcare information is often inaccessible, particularly outside of dental clinics. Some reported confusion about how to choose a dentist, especially in relation to providers’ openness to treat individuals with autism, anxiety, or other specific needs: *“Is there, for example, a list of dentists who are suitable for children with autism? I don’t know where to take my child, it’s all trial and error. If things don’t go well with one dentist, you try another, but then my child no longer wants to go.”- Amsterdam, FGD 1, citizen-participant 2.*

*4. Emotional and Psychological Barriers*

A recurring theme was the emotional toll of poor oral health, often intertwined with feelings of shame, fear of judgment, or past traumatic experiences: *“If you look at my teeth, you can see they’re very crooked. It has a huge impact on my life. I never smile because my teeth are broken, damaged, and uneven. Every time I look in the mirror, I see how bad they are. Not having the money to fix it only makes it worse.” – Rotterdam, individual interview 10, citizen who received informal no-cost dental care. “Because of the poor care I received from my parents, my teeth are in bad condition, and I’ve often had to have them pulled. Those experiences make me reluctant to go, and it also costs money I don’t really have.”- Rotterdam, individual interview 13, citizen who received informal no-cost dental care.* Several participants reported dental anxiety or general distrust in dentists: *“I go with my instinct. When the dentist started, I didn’t feel trust. The chair moved constantly, she worked quickly, and I had to pay without explanation. I felt like I was being scammed.”- Amsterdam, individual interview 5, homeless person.* To help overcome these barriers, participants emphasized the need for neutral, low-threshold opportunities to engage with dental professionals or receive dental care outside of clinical settings: *“Any simple place with a clean room, a sink, and a good chair could make a difference, if we could just come every six months to get our teeth cleaned without paying an arm and a leg.”- Amsterdam, individual interview 2, homeless person.*

*5. Competing Priorities*

Many citizens described their oral health as being deprioritized due to more pressing survival needs, such as housing insecurity, mental health challenges, or financial instability. Social workers echoed this sentiment, noting that oral health is often not integrated into the broader framework of general or social care, and that they are rarely trained or resourced to address it: *“Oral health is not really a major issue next to e.g. housing issues, until someone experiences problems.”- Rotterdam, individual interview 8, program manager social fund. “Maybe a workshop or training on oral health for social workers might be beneficial, where people can share their experiences and a professional provides information about the available options.”- Rotterdam, individual interview 6, social worker*

*6. Social Support*

Lack of both professional and informal social support was identified as a key challenge. Some participants described having no one to rely on for transportation to appointments, or for help understanding health information. Friends and peers were often in similarly vulnerable positions, making it difficult to borrow money or share resources: *“Yeah, who are we supposed to ask for money for the dentist? silence I wouldn’t know who, my friends are struggling too.”- Amsterdam, individual interview 1, recipient emergency fund.* On the other hand, participants described positive experiences with professional social support: *“My social worker knew there was an emergency fund for dental costs, so she just decided to try. Nothing to lose, right? But I really didn’t expect to receive that much money, I was very happy about it’’- Amsterdam, individual interview 1, recipient emergency fund.*

*7. Communication and Professional Behaviour*

Firstly, the way in which oral healthcare professionals communicate was seen as a critical factor. Participants emphasized the need for clear agreements about costs, the provision of consistent information, and more empathetic communication, especially during initial visits: *“Dentists should be more present in the community, more accessible, with time reserved for urgent treatments so people in pain can be seen quickly. They should be transparent, welcoming, and friendly, treat people in vulnerable situations with respect, and allow patients to maintain control over their own treatment.” – Amsterdam, FGD 2, participants 6 summarizing the main take-aways about communication and professional behaviour.* They also noted a lack of collaboration between oral health professionals and general practitioners and described consultation settings as too rushed or intimidating to ask questions.

### Co-created solutions for overcoming barriers

Participants collaboratively developed several practical ideas to address the barriers identified above. These are summarized per level of solutions (policy, practice or community) in Figure 1. The figure shows that solutions may be helpful for more than one barrier, while at the same time most barriers ask for more than one solution. Many solutions can be applied at more than one level, that is, at policy, practice and community level, although they often apply primarily to one of these. An example is communication: whilst communication in plain language is primarily a practice’s concern, efforts to address communications to vulnerable group in a way that is tailored to their needs are also a policy matter.

*Overcoming financial barriers:* Participants suggested expanding oral healthcare insurance options for low-income groups, distributing oral hygiene products through municipalities or food banks, and organizing community-based information sessions (e.g., walk-in consultations at neighborhood centers). There was also support for expanding informal care initiatives such as cost-free oral healthcare by volunteers (“Social Dentist”) and encouraging installment-based payment options in dental practices.

*Overcoming health insurance barriers:* Proposed solutions focused on strengthening citizens’ role in national advocacy to expand dental coverage and offering local support by professionals within community centers to help residents navigate complex insurance systems. Participants emphasized that current barriers extend beyond financial issues to include difficulties in understanding and comparing multiple insurance schemes. A helpful role for dental students and (oral) health professionals in explaining these matters was endorsed.

*Improving knowledge and health attitudes:* Citizens recommended integrating oral health education into school curricula and providing verbal (rather than written) information in community settings. They also suggested that professional dental associations could support vulnerable groups by providing (analogue and digital) resources such as maps of inclusive dentists trained to work with marginalized populations.

*Avoiding competing priorities:* Participants proposed embedding oral health within social care pathways and developing training programs for social workers to raise awareness and strengthen their role in early identification and referral.

*Strengthening social support:* Suggestions included establishing peer-support or buddy systems within community centers, where trained volunteers or neighbors could assist with appointment scheduling, transportation, and oral health education.

*Shorter communication lines:* Participants proposed community outreach by oral health professionals, walk-in consultations in neighborhood centers, inclusion of oral health information in general practice clinics, and training modules for dental teams on communication and working with vulnerable populations.

## Discussion

This study illustrates that people living in vulnerable urban communities face many different and often deeply rooted needs and barriers when trying to access oral healthcare. It also shows the process that has led to the co-creation of fruitful multistakeholder networks in urban communities for improvement of oral health and care. It has furthermore demonstrated that accessibility and quality of oral care is not just a financial matter. While financial barriers such as high treatment costs, limited insurance coverage, and lack of affordable self-care products were consistently prioritized by citizen participants, citizen and professional participants also voiced many non-financial challenges. They ranked these among their main concerns. These include psychosocial factors such as shame, dental anxiety, and distrust in providers, as well as practical obstacles like lack of social support, low oral health literacy, and complex healthcare navigation, all of which contribute to limited access to oral healthcare. As a result, the solutions suggested by community members and professionals went far beyond just changing insurance policies or reducing costs. Change was set into motion by the act of establishing these community groups. Solutions were not just discussed, but created: interdisciplinary connections were made, a sense of urgency for oral health care accessibility and quality was forged, and the groups felt empowered to keep this issue on both their own and local politician’s agendas. This translated into concrete actionable steps: lasting connections between the networks and Dental schools were made, policymakers got involved, NGOs got onboard to organize free of costs emergency care-days and connections with Food banks and other initiatives were made. At the National Dentists’ Day on September 27, 2025, 82 dental practices across the Netherlands provided basic emergency oral care to over 1000 individuals free of charge.^24^

Our results are in line with earlier research showing that inequalities in oral health are closely linked to broader social factors, such as income, education and living conditions.^25^, ^26^, ^27^ Our findings reflect the recent *World Health Organization Global Oral Health Status Report*, which emphasizes that despite being largely preventable, oral diseases remain highly prevalent among lower-income groups and are not sufficiently integrated into universal health coverage schemes, especially those for adults.^28^ Participants in our study described that oral healthcare was often perceived as a luxury rather than a basic health right, especially when financial resources were limited or competing needs were more urgent. This is consistent with previous qualitative research among Indian migrants in the Netherlands, which similarly reported that oral healthcare utilisation is shaped by structural and socioeconomic determinants, including insurance systems, preventive orientation of Dutch dental care, and communication-related barriers.^29^ However, that study mainly included higher-educated migrants, whereas our findings extend this evidence by capturing a broader socio-economic spectrum, including more vulnerable participants who more explicitly framed dental care as competing with other essential life priorities, thereby highlighting how financial strain and living conditions further shape perceptions of oral healthcare as a non-essential or “luxury” service.

Importantly, this study shows that community-driven solutions are realistic and achievable at the local community level. Earlier studies have already shown that community-based initiatives can be effective in improving oral health among underserved groups.^30^, ^31^, ^32^ The interventions proposed by our respondents, such as walk-in consultations in neighborhood centers, buddy systems to assist with access and understanding, maps of inclusive providers, and informal community dialogues with dental professionals, illustrate practical ways to reduce inequality in oral healthcare accessibility. These ideas align with WHO’s call to strengthen intersectoral collaboration and to adopt participatory, locally tailored approaches in public health.^28^ However, we found that oral health is often absent from the agendas of social workers, general practitioners, and community organizations who are otherwise well-positioned to identify and refer citizens in need. This finding is also in line with another study from the Netherlands that similarly reported that oral health is insufficiently prioritized within interprofessional care networks for geriatric patients, with unclear responsibilities and limited structural integration of oral health into routine collaboration between health care professionals.^33^

Beyond the DELIVER project, a further step will be to put these ideas further into practice by testing the proposed solutions in the community networks (both in cities and rural areas).^5^ At the same time, this co-created model may serve as a blueprint for other neighborhoods across the Netherlands or similar European settings seeking to improve oral health equity through community-based approaches.

To encourage meaningful change, several policy and practice implications should be considered. Oral health could be more explicitly recognized as an integral component of overall health, with implications for insurance coverage for underserved populations and its inclusion in local health policies. In addition, social workers and other frontline professionals may benefit from additional training and support to better identify oral health needs and facilitate referrals to appropriate care providers. This can be particularly relevant in contexts where more immediate concerns, such as housing or income insecurity, tend to limit attention to oral health needs. Given the role municipalities can play in promoting oral health, there is an opportunity, and arguably a responsibility for them to further support social and local health workers in fulfilling this role in a more substantive way. Finally, clarifying ownership and accountability may help strengthen the continuity of efforts and support the long-term sustainability and scaling of initiatives. In the Netherlands, no one seems fully responsible for improving access to oral healthcare. Different stakeholders often point to each other.^7^ This accountability gap could be bridged by strengthening the roles and involvement of Chief Dental Officers (CDOs), who can promote national and regional coordination, ensure policy alignment, and drive the scalability of community-based interventions.

Regarding sustainability, the project aims to progressively transfer ownership from academic partners to community stakeholders, including citizens, professionals, and municipal actors. The co-created activities are designed as a prototype community service and will be supported by a practical toolkit towards the end of the DELIVER project to facilitate adaptation and implementation in other local contexts. Sustainability will be further supported by embedding activities within existing local and national networks, as well as alignment with ongoing policy initiatives and engagement with key stakeholders such as Chief Dental Officers. The emergence and scaling of community-driven initiatives, such as National Dentists’ Day,^24^ illustrate how locally embedded efforts can evolve into sustained practice when supported by existing structures and key actors.

This study also has certain limitations that should be acknowledged. Participants were recruited through community organizations, which may have excluded individuals who are most socially isolated or who have limited trust in institutional settings. The use of convenience and snowball sampling may limit the generalizability of the findings, as the sample may not be fully representative of all individuals in vulnerable circumstances across urban settings. However, our study population still contained a wide range of citizens and professionals from a variety of backgrounds. In addition, although a diversity of perspectives was represented, power imbalances among stakeholders within focus groups may have shaped which experiences or viewpoints were expressed. Future research could therefore focus on strategies to engage more disconnected populations and examine how the proposed solutions function when implemented in practice. At the same time, the study has notable strengths. It engaged a broad range of stakeholders, including citizens, professionals, and policy actors, through co-creative and participatory approaches that fostered rich dialogue and the generation of actionable insights. The stepwise research design allowed participants to actively shape each phase of the process. Moreover, the use of creative methods such as sticky notes, posters, and storytelling helped lower participation barriers and supported the inclusion of individuals with lower literacy levels, who otherwise tend to be disregarded in research.^34^

## Conclusion

This study highlights and addresses the complex and interconnected barriers that many citizens in vulnerable urban communities face when trying to access oral healthcare. By actively involving citizens and professionals in co-creation, the DELIVER project revealed a range of practical, community-driven solutions that go beyond policy reform and speak directly to lived experience. As the project continues, these insights already help shape local actions to improve oral health accessibility. This participatory model offers a blueprint for other neighborhoods across Europe, adaptable to the local context. When people are engaged as partners, community-driven solutions become possible.

## Author contributions

**S. Begovic:** Conceptualization; Methodology; Formal analysis; Investigation; Data curation; Writing Original Draft; Visualization. **M.W. van der Linden:** Conceptualization; Methodology; Formal analysis; Investigation; Writing Review and Editing; Project administration; Supervision. **S.J. Gitz:** Conceptualization; Methodology; Data curation; Writing Review and Editing. **S. Listl:** Conceptualization; Methodology; Writing Review and Editing; Project administration; Funding acquisition. **L. Eisemann de Almeida:** Conceptualization; Writing Review and Editing. **E. Boeskov Øzhayat:** Conceptualization; Writing Review and Editing. **K. Rosing:** Conceptualization; Writing Review and Editing. **C.M.C. Volgenant:** Conceptualization; Methodology; Writing Review and Editing; Supervision. **M.H. van der Veen:** Conceptualization; Methodology; Supervision; Writing Review and Editing.

## Funding

The authors disclosed receipt of the following financial support for the research, authorship, and/or publication of this article: This study is part of the DELIVER project, which has received funding from the European Union’s HORIZON Europe research and innovation program under grant agreement 101057077: https://cordis.europa.eu/project/id/101057077. Views and opinions expressed are, however, those of the authors only and do not necessarily reflect those of the European Union. Neither the European Union nor the granting authority can be held responsible for them.

## Conflict of interest

None disclosed.
